# Ocular and Plasma Pharmacokinetics of Enavogliflozin Ophthalmic Solution in Preclinical Species

**DOI:** 10.3390/ph17010111

**Published:** 2024-01-13

**Authors:** Mingui Jang, Minsung Kang, Eunseok Lee, Dongseong Shin

**Affiliations:** 1 Center of Development, Daewoong Therapeutics Inc., Hwaseong-si 18469, Gyeonggi-do, Republic of Korea; jangalsrnl@daewoongrx.co.kr (M.J.); esl0409@daewoongrx.co.kr (E.L.); 2 Center of Nonclinical Drug Evaluation, Daewoong Therapeutics Inc., Hwaseong-si 18469, Gyeonggi-do, Republic of Korea; mkang@daewoongrx.co.kr; 3 Department of Clinical Pharmacology and Therapeutics, Gil Medical Center, Gachon University College of Medicine, Incheon 21565, Republic of Korea

**Keywords:** enavogliflozin, SGLT2 inhibitor, ocular pharmacokinetics, diabetic retinopathy, diabetic macular edema

## Abstract

An enavogliflozin ophthalmic solution (DWRX2008) is being developed to treat diabetic retinopathy and macular edema. This study evaluated the ocular distribution and plasma pharmacokinetics (PKs) of enavogliflozin in animal species. A sample of [^14^C] enavogliflozin was ocularly administered to two rabbits per time point at single doses of 600 μg/eye to evaluate ocular PK, which was evaluated using autoradiography until 48 h post-dose. Plasma concentrations after ocular administration in six rabbits, three rats, and three beagle dogs with single doses of 400 μg, 25 μg, and 100 μg, respectively, were investigated for 24 h. The retinal concentration of [^14^C] enavogliflozin reached C_max_ at 2.0 h with an elimination half-life of 32.5 h, which remained above the IC_50_ value of sodium-dependent glucose transporter 2 until 24 h post-dose. In the plasma of rabbits, the fastest T_max_ of 0.5 h and a 3.6 h half-life were observed among animal species. The relative bioavailability in rabbits after ocular administration was 3.4 compared to oral administration. Ocular administration of enavogliflozin could be a potential therapeutic route for diabetic retinal complications, based on relative bioavailability and effective delivery to the posterior ocular segment. DWRX2008 would be applicable to humans with favorable PK profiles and minimal systemic adverse effect.

## 1. Introduction

Retinal diseases including diabetic retinopathy (DR), age-related macular degeneration, and glaucoma are a growing concern in the aging population [1]. DR is a common and serious microvascular complication of diabetes, and its incidence increases with diabetes progression; a previous epidemiological study found that after 5 and 10 years of diabetes diagnosis, the incidence of DR was approximately 25 and 60%, respectively [2]. DR is a progressive microvascular alteration associated with chronic hyperglycemia, resulting in retinal ischemia, permeability, and neovascularization as well as macular edema [2]. Diabetic macular edema (DME), an advanced complication of DR, is defined as the thickening of the retina or the presence of hard exudates within one disk diameter of the center of the macula [3]. The blood–retinal barrier breakdown and accumulation of fluid and molecules can induce retinal thickening at any stage of non-proliferative and proliferative diabetic retinopathy [4]. These diabetic retinal complications are the main cause of visual impairment and irreversible blindness in middle aged and elderly people [2]. Furthermore, DR and DME implies the existence of other microangiopathies based on its correlation with macrovascular damages [5]. As untreated diabetic retinal complications can cause vision loss, early detection and timely treatment are vital. The progression of DR and DME can be effectively delayed by tightly controlling blood glucose levels and blood pressure [5]. Standard pharmacological treatments for diabetic retinal complications include intravitreal injections of anti-vascular endothelial growth factor agents, often accompanied by laser photocoagulation and vitrectomy [6]. Although anti-vascular endothelial growth factor therapy is an effective treatment option, it has several shortcomings, such as frequent injection, high cost, and substantial retinopathy progression [7]. At present, effective treatments and novel drugs for the treatment of early-stage diabetic retinal diseases are limited, owing to the difficulty of drug delivery to the retina and the economic burden associated with such treatments [1].

Sodium-dependent glucose cotransporters (SGLTs) are active glucose transporters [8]. When sodium binds at the extracellular surface, the outside gate of proteins open and trap outside sugar [8]. Then, the protein flips over to the intracellular side, and sodium and glucose are released into the cell interior [8]. Six SGLT isoforms have been reported in humans [8]. Among the SGLTs, SGLT1 is mainly found in the intestine, trachea, kidneys, heart, and brain [8]. SGLT2 is reportedly localized in renal tubular cells, islet α-cells, prostatic and pancreatic cancer cells, glomerular mesangial cells, and retinal pericytes [6]. As these two SGLTs have a critical role in the glucose absorption in the kidney and intestinal tract, novel therapeutic targets have been suggested via the blockade of transporters [8].

SGLT2 inhibitors are a new treatment option for type 2 diabetes mellitus and exhibit their effects by inhibiting the reabsorption of glucose in the proximal renal tubules and increasing urinary glucose excretion [7]. Additionally, SGLT2 inhibitors report weight reduction and renoprotection benefits [6,7]. Ocular SGLTs are distributed throughout the lens and retina [2]. In the retina, SGLT2 plays a role in transporting glucose and sodium to the pericytes [6]. As excessive sodium and glucose entry via SGLT2 leads to pericyte swelling and loss, SGLT2 inhibitors exert therapeutic effects by removing excessive glucose from the retinal microcirculation, reducing oxidative stress and low-grade inflammation, and restoring insulin signaling [5,6]. Moreover, SGLT2 inhibitors also enhance treatment efficacy by improving the factors involved in the pathogenesis of diabetes including hyperglycemia, hypertension, and hyperlipidemia [2]. In addition to these benefits, the protection of ocular tissues including the blood–retinal barrier, fundus capillaries, and optic nerve, is a possible mechanism through which SGLT2 inhibitors treat DR [2]. In the previous study, marked regression of DME was reported after the treatment of SGLT2 inhibitors compared to other antidiabetic agents that showed the possibility of beneficial or neutral effects on diabetic retinal disease through tight glucose control and neuroprotective effect [9].

DWRX2008 is a topical ophthalmic solution containing enavogliflozin, an SGLT2 inhibitor. Enavogliflozin selectively and reversibly inhibits SGLT2 more than SGLT1 with a much lower IC_50_ value [10]. In animal studies, DWRX2008 has shown promising therapeutic potential for reducing retinal vascular permeability (Daewoong Therapeutics, DWRX2008-IB-01 Investigator’s brochure, data on file). Additionally, ocular safety after multiple ocular instillations in rabbits did not show significant toxicity (Daewoong Therapeutics, DWRX2008-IB-01 Investigator’s brochure, data on file). Drug delivery to the posterior eye remains a major challenge in the development of effective ocular therapeutic agents because of the complexity and uniqueness of ocular anatomy and physiology [11]. Therefore, understanding ocular pharmacokinetics (PK) is essential for drug design and development [11]. Ocular PKs have rarely been studied in humans, owing to ethical concerns and the PK effects of invasive sampling from the human eye [1]. Consequently, ocular PKs depend on animal models [1].

In this study, the ocular distribution of enavogliflozin in the conjunctiva, cornea, aqueous humor, and other intraocular tissues, and its absorption into the systemic circulation were investigated after a single topical administration of DWRX2008 eye drops into the eyes of rabbits.

## 2. Results

### 2.1. Ocular Distribution of Enavogliflozin

**Figure 1:**
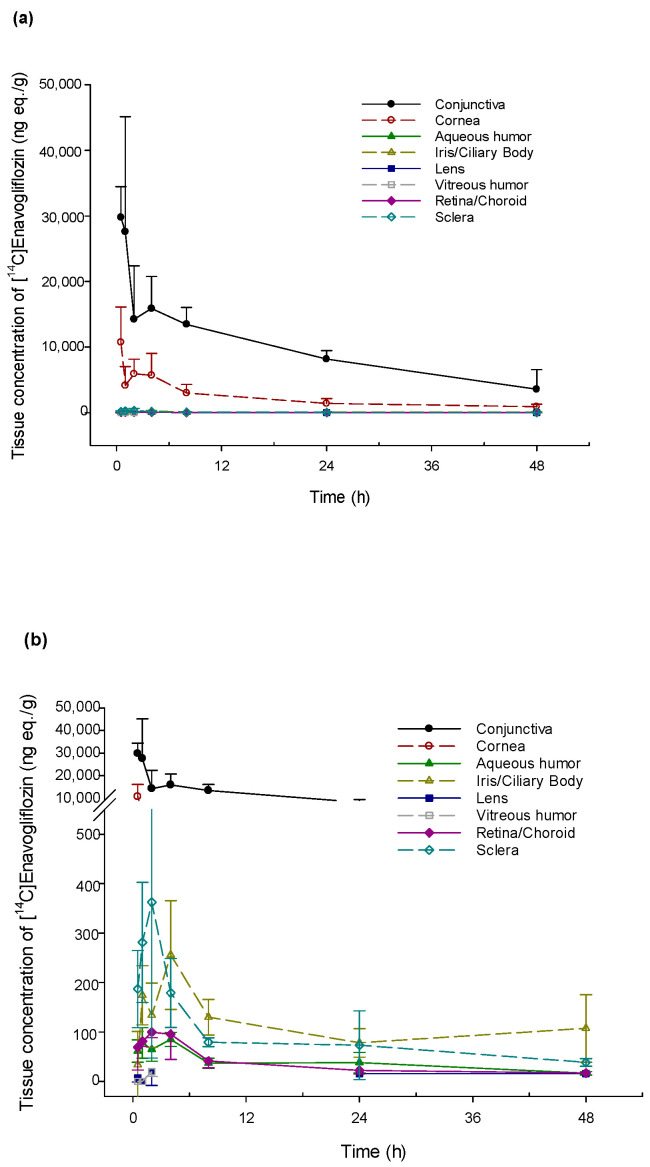
Radioactivity concentration–time profile in ocular tissues after single topical ocular administration of [^14^C] enavogliflozin in male New Zealand White rabbits. (**a**) Ocular tissues including the conjunctiva and cornea. (**b**) Specific ocular tissues except for conjunctiva and cornea.

PK parameters were estimated using the mean radioactivity concentration in both eyes at each time point because each eye could be considered as an individual [12]. After a single ocular administration of DWRX2008, the radioactivity level of [^14^C] enavogliflozin in various ocular tissues revealed a higher exposure of [^14^C] enavogliflozin in the conjunctiva and cornea (Figure 1). In the conjunctiva and cornea, the concentration of [^14^C] enavogliflozin rapidly reached the maximum concentration (C_max_) after 0.5 h of administration. Based on the mean radioactivity concentration, the half-lives of [^14^C] enavogliflozin in the conjunctiva and cornea were 20.7 and 18.0 h, respectively. In the retina, the target tissue of the disease, C_max_ and the area under the concentration–time curve (AUC_0–t_) were 99.5 ng eq/g and 1594.2 ng eq·h/g, respectively, with a half-life of 32.5 h. A T_max_ of 2 h was observed in the retina. Radioactivity was barely detectable in the optic nerve. The PK parameters of ocular distribution are summarized in Table 1.

### 2.2. Blood Exposure to Enavogliflozin

Systemic exposure to radiolabeled [^14^C] enavogliflozin was investigated in two independent rabbit groups. In one group, serial blood collection from three rabbits was performed for 48 h after a single ocular administration of DWRX2008 (600 μg/eye). The C_max_ and AUC_0–t_ of radioactivity concentration in the plasma were 13.0 ± 1.7 ng eq/g and 42.8 ± 21.3 ng eq·h/g, respectively, and the T_max_ was 1.0 h. The estimated elimination half-life in one subject was 21.5 h. In the other group, the rabbits were euthanized, and plasma and whole blood with ocular tissues were obtained at various time points up to 48 h after a single ocular dose of DWRX2008 (600 μg/eye) (Table 1). In plasma, the C_max_ was 17.6 ng eq/g at 1.0 h post-dose and AUC_0–t_ was 102.3 ng eq·h/g, with a half-life of 9.9 h. Compared with plasma, [^14^C] enavogliflozin exhibited lower concentrations and faster elimination in whole blood; the C_max_ was 12.9 ng eq/g and the AUC_0–t_ was 65.5 ng eq·h/g, with a half-life of 5.7 h. The C_max_ and AUC_0–t_ of [^14^C] enavogliflozin in whole blood were 73.4% and 64.0% of their corresponding values in the plasma, with the same T_max_. Elimination in the ocular tissues was slower, compared to systemic elimination.

**Figure 2:**
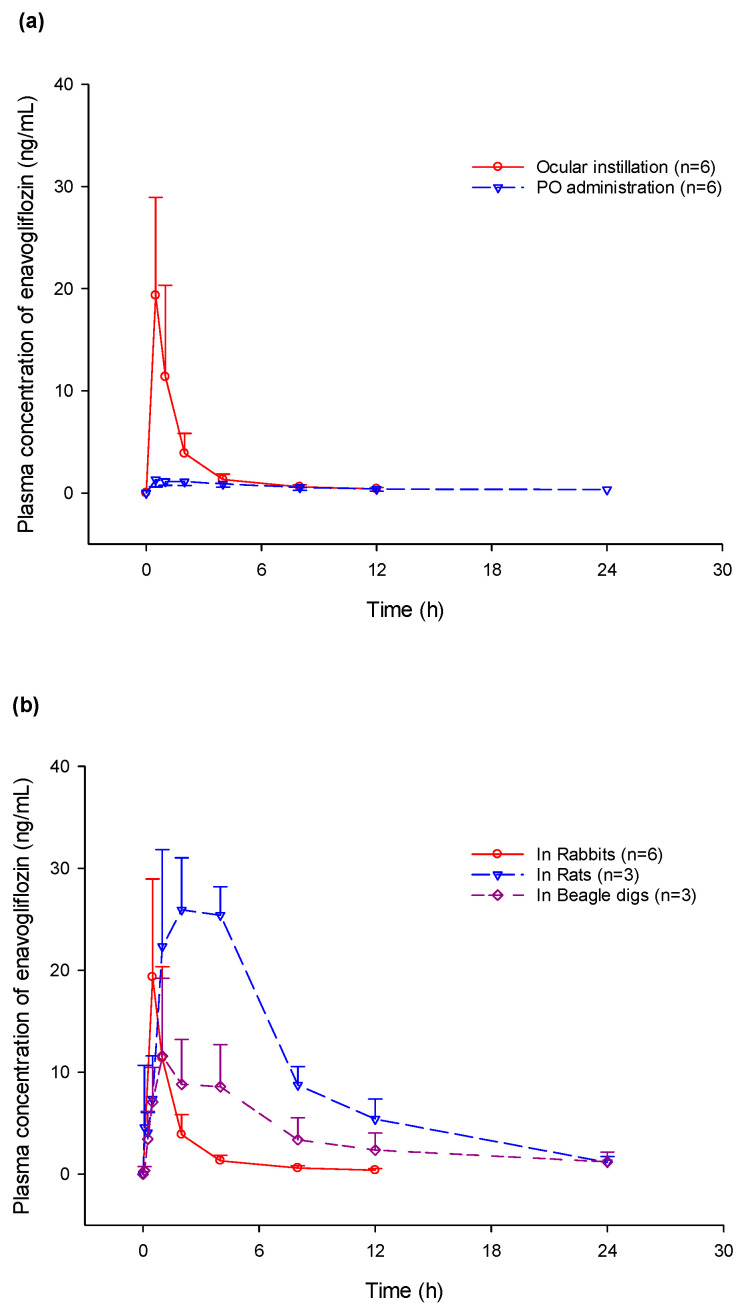
Plasma concentration–time profile in multiple animal species. (**a**) Comparison of plasma enavogliflozin concentration between ocular and oral administration of 400 μg/head; (**b**) Comparison of plasma concentration among rats with a dose of 25 μg on a single eye, rabbits with a dose of 200 μg on both eyes, and beagle dogs with a dose of 100 μg on a single eye.

Plasma concentrations after a single ocular administration were evaluated in various animal groups including rabbits, rats, and beagle dogs (Figure 2). In rats, the median T_max_ of enavogliflozin in the plasma occurred at 4.0 h, with a mean half-life of 5.4 h. In beagle dogs, the plasma concentration of enavogliflozin reached its peak concentration at 1.0 h and was eliminated at a mean half-life of 10.6 h (Table 2).

## 3. Discussion

Enavogliflozin is a novel selective SGLT2 inhibitor with a potential suppressive effect on hyperglycemic vascular leakage in the retina (Daewoong Therapeutics, DWRX2008-IB-01 Investigator’s brochure, data on file). For the development of enavogliflozin as an ocular agent, the evaluation of a dosing method which can deliver the drug efficiently to the target site is necessary. Topical, systemic, intraocular, and periocular approaches can be applied for drug delivery to the posterior segment [13]. Topically applied drugs have difficulty in yielding sufficient therapeutic levels owing to significantly limited delivery [13]. Systemic administration can overcome these barriers to achieve an effective concentration in the retina and vitreous. However, large systemic doses are often associated with adverse effects including diabetic ketoacidosis, bone fracture, cancer, and genitourinary tract-associated SGLT2 inhibitors [14]. Consequently, despite several benefits, such as good compliance, self-administration, and noninvasive methods, topical ocular administration has significant challenges owing to tear dilution, turnover rate, anatomical barriers, low bioavailability, and systemic adverse effects [15]. It is essential to understand preclinical PK profiles during drug development to expand therapeutic utility and minimize systemic drug exposure. However, for diabetic retinopathy and macular edema, PK profiles from various animal models after ocular administration of enavogliflozin are still insufficient to assess its intraocular distribution or systemic exposure.

Rabbits have served as a clinically predictable animal model for the intravitreal PKs of drug delivery to the retina and/or choroid, based on small differences in the physiological and PK parameters between humans and rabbits [1]. PK parameters including half-life, showed a good correlation and reliable rabbit-to-human translation [1,12]. In this study, ocular distribution of enavogliflozin in rabbits was investigated. Ocular drugs are generally absorbed into intraocular tissues through corneal and non-corneal (conjunctiva/scleral) routes [16]. The corneal route is generally favorable for lipophilic and small-molecule drugs, and is considered the most common pathway for topical ocular drug absorption [17]. In contrast, the noncorneal route which consists of highly vascularized tissue, is important for large-molecule and hydrophilic drugs [17]. The corneal route which involves direct delivery to the anterior segment, leads to a high drug concentration in the cornea, aqueous humor, and iris–ciliary body [17]. The noncorneal route which traverses the conjunctiva and sclera, contributes to drug delivery to the choroid and retina [17]. Conjunctival exposure through the non-corneal pathway results in a low drug concentration in the aqueous humor, in contrast to the corneal pathway, which shows a typical drug distribution pattern with concentrations following the order: cornea > aqueous humor, iris, ciliary body > sclera > lens > retina/choroid and vitreous humor [16]. Ocular PKs can be affected by the interaction between melanin binding and systemic clearance, plasma protein binding, cellular permeation, efflux proteins including P-glycoprotein, multidrug resistance protein, and breast cancer resistance protein, pH partitioning, and tissue binding [18]. Strong melanin-binding drugs have exhibited drug accumulation and prolonged retention in melanin-containing tissues, including the choroid, sclera, and retina [18,19].

Enavogliflozin is a small-molecule (molecular weight: 446.92 g/mol) and lipophilic (partition coefficient [Log P]: 2.13) drug. After ocular administration in both eyes of the rabbits, the tissue concentration of enavogliflozin did not differ significantly between the left and right eyes in the overall tissue (*p* = 0.242) or retina (*p* = 0.155). The mean concentrations of enavogliflozin on both sides of the ocular system were assessed to analyze the PK profiles. Considering the physicochemical properties of enavogliflozin, the corneal pathway may be preferable for access into the intraocular tissues. However, in the PK profiles of ocular tissues, the AUC_0–t_ was the highest in the conjunctiva. The T_max_ in the sclera and retina were similar and faster than that in the aqueous humor, with a tendency towards greater AUC_0–t_ in the sclera and retina. Although concentration–time profiles were not obtained in the same subject, these intraocular PK profiles suggest that the non-corneal route might contribute to the delivery of enavogliflozin to the posterior segments, dominantly [17,20]. In addition, enavogliflozin is considered a moderate-affinity melanin binder with an approximately 33% melanin binding capability, which may lead to longer retention at concentrations close to the C_max_ in the retina. Additionally, the mean retina/plasma (R/P) drug concentration ratios for 1, 3, and 6 h after administration in rats were 3.3 (range: 0.6–6.6), 1.3 (range: 0.2–2.2), and 2.6 (range: 1.4–3.3), respectively. These ratios indicate that enavogliflozin is distributed more readily to the retina. Based on the molecular weight of enavogliflozin, the concentrations in the retina of rabbits after a single ocular administration of 600 μg were expected to be higher than the inhibitory concentration against hSGLT2 (IC_50_: 0.43 nM) until 24 h. However, the plasma concentrations of enavogliflozin were lower than the IC_50_ values in various vital organs. The IC_50_ values were reported as 44.9 μM for hERG assay and no effect for other systems (Daewoong Therapeutics, DWRX2008-IB-01 Investigator’s brochure, data on file). Therefore, there is a low possibility of adverse effects via systemic circulation after ocular administration.

Regarding systemic absorption after ocular administration, plasma concentration could develop through intestinal absorption of enavogliflozin through the nasolacrimal duct from the precorneal area, and elimination into the systemic circulation via diffusion from ocular tissues [19,21]. In these studies, the systemic PK profiles after ocular administration were evaluated in rats, rabbits, and beagle dogs. Considering the fast T_max_ in the plasma concentration–time profiles, systemic exposure was possibly affected by non-corneal absorption by the conjunctival vasculature and lacrimal drainage [19]. The fastest T_max_ and rapid elimination were observed in rabbits. Species–specific metabolism and systemic clearance of enavogliflozin have been previously reported [10]. Cytochrome P450 (CYP) enzymes CYP 3A4 and CYP 2C19, and UDP-glucuronosyltransferases (UGTs) UGT 1A4 and UGT 2B7 are primarily involved in hepatic metabolism [10]. CYP and UGT enzymes mainly contribute to Phase I and II metabolisms, which are involved in the elimination of approximately 50% and 10% of the commonly used drug, respectively [22]. Although the CYP and UGT super families possess highly conserved regions of amino acid residues, interspecies differences in their primary amino acid sequences have been reported [22]. Minor changes in the amino acid sequences at the active sites of CYP enzymes can cause a wide variation of substrate specificity and catalytic activity of drug-metabolizing enzymes [22]. Therefore, differences in CYP or UGT isoforms can have significant effects on the differences in drug metabolism across varying species [22].

Both the plasma and retinal concentrations of enavogliflozin were measured after oral administration in rabbits. The absorption between the ocular and oral routes of administration was compared. Systemic absorption after ocular administration was greater than that after oral administration, with a ratio of approximately 3.4. In contrast, the retinal enavogliflozin concentration at the same oral dose was below the lower limit of quantification. Eventually, the drug concentration in the targeted tissue with ocular administration was promising, whereas the therapeutic effect via systemic circulation would be ineffective.

This study had several limitations including that it involved only a small number of animals and independent animal groups at each time point after a single dose. Furthermore, PK results were obtained based on the mean concentrations although there was no difference between the sides of the eye. Because plasma PK profiles were investigated at different dose levels in various animal species, evaluation of consistent pharmacokinetic tendency was limited. Consequently, large-scale clinical research using multiple doses is required in human subjects to reflect the physiology and dosing methods in humans. In this study, ocular PKs in rabbits revealed therapeutic levels of enavogliflozin at the target site and limited drug exposure in systemic circulation. Based on these ocular distribution and systemic PKs, DWRX2008 will be applied to human eyes in clinical trials with favorable PK profiles and minimal safety concerns. The ocular administration of enavogliflozin could be a potential therapeutic strategy for the treatment of diabetic retinopathy.

## 4. Materials and Methods

### 4.1. Ocular Distribution Study

#### 4.1.1. Test Formulation and Analytical Conditions for Ocular PK

The [^14^C] enavogliflozin used in this study, with a specific radioactivity of 4.72 MBq/mg, was synthesized and radiolabeled by Curachem (Cheongju, Republic of Korea). The 98.3% radiochemical purity of [^14^C] enavogliflozin and 99.4% chemical purity of non-radiolabeled enavogliflozin were established through high-performance liquid chromatography (Agilent 1100 series; Agilent Technologies, Santa Clara, CA, USA) equipped with the Berthold FlowStar LB513 (Berthold Technologies, Württemberg, Germany) radioactivity detector, using a Capcellpak MG C18 column (4.6 mm × 250 mm, particle size: 5 μm; Osaka Soda Co. Ltd., Osaka, Japan). The mobile phase consisted of 0.1% phosphoric acid in water (phase A) and acetonitrile (phase B) at a flow rate of 1.0 mL/min and column temperature of 25 °C.

#### 4.1.2. Animals and [^14^C] Enavogliflozin Administration for Ocular PK

We used 9-month-old male rabbits (Envigo, Denver, PA, USA) weighing 3.1–3.7 kg in the PK studies involving a single topical ocular administration of [^14^C] enavogliflozin. The rabbits were acclimated for a minimum of seven days and housed individually under a 12/12 h light/dark cycle. The rabbits were fed commercial rabbit chow (PMI Certified Rabbit Diet #5325) once daily and provided ad libitum access to water via an automatic watering system.

The dosing formulation was prepared one day before it was administered (Day-1). The appropriate quantity of radiolabeled and non-radiolabeled test formulation (if required) was combined with the vehicle to meet the dose level (~77 μCi/eye). DWRX2008 is an ophthalmic solution prepared by mixing enavogliflozin and the vehicle (70 mg/mL polyoxyl stearate, 20 mg/mL polysorbate 80, 5 mg/mL vitamin E polyethylene glycol succinate, 16 mg/mL glycerin, 0.156 mg/mL monobasic sodium phosphate, and 0.028 mg/mL sodium hydroxide in distilled water). A single volumetric dose of 30 μL was administered to each eye (600 μg/eye, 1200 μg/head).

#### 4.1.3. Ocular Distribution by Autoradiography

Two animals were euthanized by Euthasol^®^ administration into the marginal ear vein at each predetermined time point (0.5, 1, 2, 4, 8, 24, and 48 h) post-instillation. Subsequently, their heads were frozen in a hexane/dry-ice bath and embedded in a 5% carboxymethylcellulose matrix. The embedded heads were sectioned at 30 μm thickness with less than 20% variation.

### 4.2. Plasma PK Study

#### 4.2.1. Animals and DWRX2008 Administration for Plasma PKs

We used 7-week-old male Brown Norway rats (Central Lab. Animal Inc., Seoul, Republic of Korea) weighing 140–180 g [23]. The rats were acclimated for a minimum of 7 days. For the evaluation of plasma PKs after ocular administration, a single dose of 5 μL of DWRX2008 0.5% (25 μg of enavogliflozin) was topically applied on the left eye. One-year-old male Ridglan beagle dogs (JOHN Bio Co., Nantong, China) weightng 8–10 kg were chosen [23]. The beagle dogs experienced a withdrawal period of over one month. For the evaluation of plasma PKs after ocular administration, a single dose of 20 μL of DWRX2008 0.5% (100 μg of enavogliflozin) was ocularly administered on the left eyes. In rats and beagle dogs, DWRX2008 was administered to the left eye for evaluation of safety and systemic absorption. Male New Zealand White rabbits (Samtako Biokorea, Osan, Republic of Korea) weighing 2.0–2.5 kg were used in PK studies after eye drop instillations [23]. The rabbits were acclimated for 7 days. For the evaluation of plasma PKs after ocular and oral administration, a single DWRX2008 2% dose of 10 μL (400 μg of enavogliflozin per head) was topically applied to each eye and 2 mL of enavogliflozin (400 μg per head) was orally administered. The animal subjects were housed individually in cages (two rats per cage) under a 12/12 h light/dark cycle (150–200 Lux) with a temperature of 23 ± 3 °C and relative humidity of 50 ± 20%. The animals were fed a standard diet ad libitum. These studies are summarized in Table 3.

#### 4.2.2. Determination of Plasma Enavogliflozin Concentration

A total of three rats were sampled 10 times at 0 (pre-dose), 0.08, 0.25, 0.5, 1, 2, 4, 8, 12 and 24 h post-dose. The blood was centrifuged at 5000 rpm for 10 min at 4 °C and the separated plasma was frozen and stored at −80 °C until analysis. For the analysis of enavogliflozin, 50 μL of plasma sample was mixed with 500 μL of *tert*-butyl methyl ether and 10 μL of internal standard ([D]DWP16001). After vortexing for 2 min, the mixture was centrifugated at 14,000 rpm for 5 min to obtain the supernatant. The 400 μL of supernatant was dried with N_2_ evaporator at 50 °C. The dried residue was dissolved in 100 μL methanol. After centrifugation at 14,000 rpm for 5 min, 3 μL of sample was analyzed by LC-MS [24]. Three beagle dogs were sampled at 0 (pre-dose), 0.08, 0.25, 0.5, 1, 2, 4, 8, 12 and 24 h post-dose. After centrifugation, 200 μL of plasma sample, 100 μL of deionized water, 800 μL of ethyl acetate and 20 μL of internal standard ([D]DWP16001) were mixed. After vortexing at 1500 rpm for 5 min, the mixture was centrifugated at 13,000 rpm for 10 min. The 850 μL of supernatant was dried. Finally, 3 μL of sample was analyzed by LC-MS [24]. Pharmacokinetic blood samples in six rabbits were obtained at 0.5, 1, 2, 4, 8, 12 and 24 h post-dose. After centrifugation at 5000 rpm for 10 min at 4 °C and freezing under −80 °C, 100 μL of plasma was mixed with 50 μL of deionized water, 600 μL of ethyl acetate and 20 μL of internal standard ([D]DWP16001). After vortexing for 5 min, the mixture was centrifugated at 13,000 rpm for 10 min to obtain the supernatant. The 650 μL of supernatant was dried and reconstructed by 50 μL of mobile phase. Finally, 3 μL of sample was analyzed by LC-MS [24]. Pharmacokinetic analysis was performed in a Waters ACQUITY UPLC system (Waters Corp., Milford, MA, USA) coupled to a triple quadrupole mass spectrometer (AB Sciex Triple Quad 5500+ System, AB Sciex Corp., Concord, ON, Canada). Chromatographic separation was performed at 45 °C using a Kinetex C18 column (150 × 2.1 mm, 1.7 μm; Phenomenex, Torrance, CA, USA). The mobile phase in the analysis of rats consisted of 10 mM ammonium acetate in water (phase A) and 0.1% formic acid in acetonitrile (phase B) at a flow rate of 0.4 mL/min. The detection range was 1.0–1000 ng/mL. In the analysis of beagle dogs and rabbits, the mobile phase consisted of 0.1% formic acid in 2 mM ammonium formate buffer (phase A) and 0.1% formic acid in acetonitrile (phase B) at a flow rate of 0.4 mL/min. The detection range was 0.2–50 ng/mL for beagle dogs and rabbits.

### 4.3. PK Analyses

The primary PK parameters for concentrations of enavogliflozin were explored using a noncompartmental method of Phoenix WinNonlin 8.3 (Certara, L.P., Princeton, NJ, USA). The C_max_ and T_max_ were directly accepted from observed data. The AUC_0–t_ was calculated using the linear trapezoidal method based on the mean radioactivity concentration of both eyes for ocular PKs in rabbits and the linear-up/log-down trapezoidal method for plasma PKs in rats, beagle dogs, and rabbits.

## Figures and Tables

**Table 1:** Ocular pharmacokinetic parameters of radioactivity after single ocular administration of [^14^C] enavogliflozin at a dose of 600 μg/eye in male New Zealand White rabbits.

		T_max_ (h)	t_1/2_ (h)	C_max_ (ng eq./g)	AUC_0–t_ (ng eq.·h/g)	AUC_inf_ (ng eq.·h/g)
Ocular tissues	Conjunctiva	0.5	20.7	29,794.0	445,986.1	553,328.1
	Cornea	0.5	18.0	10,725.8	104,518.8	128,946.2
	Aqueous humor	4.0	32.7	85.0	1771.9	2550.0
	Iris	4.0	204.9	255.8	5263.2	36974.5
	Lens	2.0	NC	19.5	787.8	NC
	Sclera	2.0	36.8	362.3	4102.3	6135.5
	Vitreous body	2.0	NC	19.5	9.8	NC
	Retina	2.0	32.5	99.5	1594.2	2392.0
Plasma		1.0	9.9	17.6	102.3	249.7
Whole blood		1.0	5.7	12.9	65.5	110.9

Data were obtained using autoradiography (*n* = 2 at each time point, *n* = 14 (total number of rabbits)). AUC_0–t_, area under the concentration–time curve; AUC_inf_, area under the concentration–time extrapolated to infinity; NC, not calculated.

**Table 2:** Plasma pharmacokinetic parameters in multiple animal species after single ocular administration or oral administration in only rabbits.

	Dose (μg)	T_max_ (h)	t_1/2_ (h)	C_max_ (ng/mL)	AUC_0–t_ (ng·h/mL)	AUC_inf_ (ng·h/mL)	V_d_/F (L)	CL/F (L/h)
Rat (*n* = 3)	25	4.0 [1.0, 4.0]	5.4 ± 0.7 (12.9)	28.2 ± 4.5 (16.1)	208.2 ± 38.1 (18.3)	217.5 ± 43.6 (20.0)	0.9 ± 0.1 (7.2)	0.12 ± 0.02 (20.2)
Rabbit (Ocular administration) (*n* = 6)	400 (both eye)	0.5 [0.5, 0.5]	3.6 ± 1.0 (27.2)	19.3 ± 9.6 (49.7)	29.5 ± 15.3 (52.0)	31.4 ± 15.6 (49.6)	83.5 ± 56.2 (67.3)	15.0 ± 5.9 (39.8)
Rabbit (Oral administration) (*n* = 6)	400	0.8 [0.5, 4.0]	6.8 ± 4.0 (58.4)	1.6 ± 0.5 (28.4)	8.7 ± 3.6 (42.2)	12.7 ± 5.7 (45.1)	297.0 ± 87.5 (29.5)	35.5 ± 12.6 (35.3)
Beagle dog (*n* = 3)	100	1.0 [1.0, 1.0]	10.6 ± 2.6 (24.8)	11.6 ± 7.6 (65.9)	87.9 ± 52.2 (59.4)	108.6 ± 70.6 (65.0)	20.7 ± 16.5 (79.7)	1.5 ± 1.4 (92.1)

Data are presented as mean ± standard deviation (% CV) and median [min, max] for T_max._ AUC_0–t_, area under the concentration–time curve; AUC_inf_, area under the concentration–time extrapolated to infinity; V_d_/F apparent volume of distribution; CL/F, apparent clearance.

**Table 3:** Summary of preclinical pharmacokinetic studies.

Measurement	Species	Route of Administration	Number of Animals	Number of Time Points	Dose
Ocular and blood concentration of [^14^C] enavogliflozin	Rabbit	Ocular	14 (2 per time point)	7	1200 μg (600 μg/eye, both eyes)
Plasma concentration of enavogliflozin	Rabbit	Ocular	6	7	400 μg (200 μg/eye, both eyes)
	Rabbit	Oral	6	7	400 μg
	Rat	Ocular	3	10	25 μg, left eye
	Beagle dog	Ocular	3	10	100 μg, left eye

## Data Availability

The datasets generated during the current study are available from the corresponding author on reasonable request.
